# Primary Pleural Lymphoma in an Immune-Competent Patient: A Diagnostic and Therapeutic Challenge

**DOI:** 10.3390/jpm15050162

**Published:** 2025-04-23

**Authors:** Carlos Silva Paredes, Juan Lauretta, Marien Semprun, Diego Rivera-Porras, María Elena Viloria, Valmore Bermúdez

**Affiliations:** 1 Universidad del Zulia, Facultad de Medicina, Departamento de Ciencias Fisiológicas, Maracaibo 4001, Venezuela; 2 Universidad del Zulia, Facultad de Medicina, Postgrado de Cirugía de Tórax, Maracaibo 4001, Venezuela; jflauretta@gmail.com; 3 Universidad del Zulia, Facultad de Medicina, Maracaibo 4001, Venezuela; dramariensemprun@gmail.com; 4 Universidad de la Costa, Departamento de Productividad e Innovación, Barranquilla 080001, Atlántico, Colombia; drivera23@cuc.edu.co; 5 Universidad del Zulia, Facultad de Medicina, Instituto de Investigaciones Biológicas, Maracaibo 4001, Venezuela; mariaeviloria@gmail.com; 6 Universidad Simón Bolívar, Facultad de Ciencias de la Salud, Centro de Investigaciones en Ciencias de la Vida, Barranquilla 080001, Colombia

**Keywords:** primary pleural lymphoma, video-assisted thoracoscopy, immunohistochemistry, R-CHOP chemotherapy, pleura

## Abstract

**Background:** Primary pleural lymphoma is a rare disease posing diagnostic and therapeutic challenges. **Case presentation:** We present a 65-year-old woman with dyspnoea, cough, and asthenia, with no significant past medical history. Chest X-ray and computed tomography showed extensive right pleural effusion. Video-assisted thoracoscopy demonstrated multiple pleural nodules, while pleural fluid analysis revealed a lymphocytic exudate, and finally, a primary pleural lymphoma diagnosis was confirmed by immunohistochemistry analysis in pleural nodules biopsy. **Discussion:** In this regard, eight cycles of chemotherapy with cyclophosphamide, doxorubicin, vincristine, dexamethasone, and rituximab were indicated, and after one year of follow-up, complete clinical and radiological remission was observed. **Conlusions:** We conclude that video-assisted thoracoscopy with an appropriate histopathological examination remains the gold standard for diagnosis, while R-CHOP chemotherapy plus rituximab may represent a highly effective therapeutic choice.

## 1. Introduction

Primary pleural lymphoma (PPL) is an uncommon neoplasm originating from the pleura without evidence of lymphatic disease at other sites [1], posing diagnostic and therapeutic challenges due to its variable presentation, non-specific clinical symptoms, and limited information about the molecular biology of this condition [2]. PPL is a rare cause of pleural effusion [3,4], accounting for 2.4% of primary chest wall tumours and 7% of lymphoma cases [5]. Although this condition is uncommon, it should be considered in the differential diagnosis of exudative pleural effusions, as it tends to appear in body cavities such as the pleural space, pericardium, and peritoneum, and early diagnosis and prompt treatment are crucial for improving the prognosis [6,7,8,9,10,11,12,13].

PPL frequently affects immunocompromised individuals and may also affect individuals in regions with human herpesvirus 8 (HHV-8) [14] endemic distribution. Thus, Factors like human immunodeficiency virus and Epstein–Barr virus infections have been associated with specific PPL subtypes, particularly marginal zone B-cell lymphomas [10,15,16]. In this regard, some evidence indicates that chronic pleural inflammation triggers B-lymphocyte proliferation stimulation [17]. Thus, empyema [18,19,20], autoimmune conditions such as Sjögren’s syndrome [21,22], asbestos exposure [23], and prior pleural trauma [24] have also been linked to PPL. These factors, alongside environmental and genetic factors, may influence the histological subtypes reported to date, such as diffuse large B-cell lymphomas, T-cell lymphomas, and the marginal zone B-cell lymphoma (MALT) being the most common [10].

A personalised approach is paramount for optimising clinical outcomes in diseases such as primary pleural lymphoma. Given the marked heterogeneity in clinical and molecular features among patients, integrating advanced diagnostic techniques—including immunohistochemistry and genomics—with tailored therapeutic strategies can significantly improve diagnostic precision and therapeutic efficacy.

This work aims to describe a PPL clinical case in an immunocompetent patient, highlighting the pharmacologic therapy and illustrating how integrating these tools enabled a tailored management strategy to the patient’s specific characteristics, resulting in complete remission and a favourable follow-up.

## 2. Case Presentation

**Figure 1:**
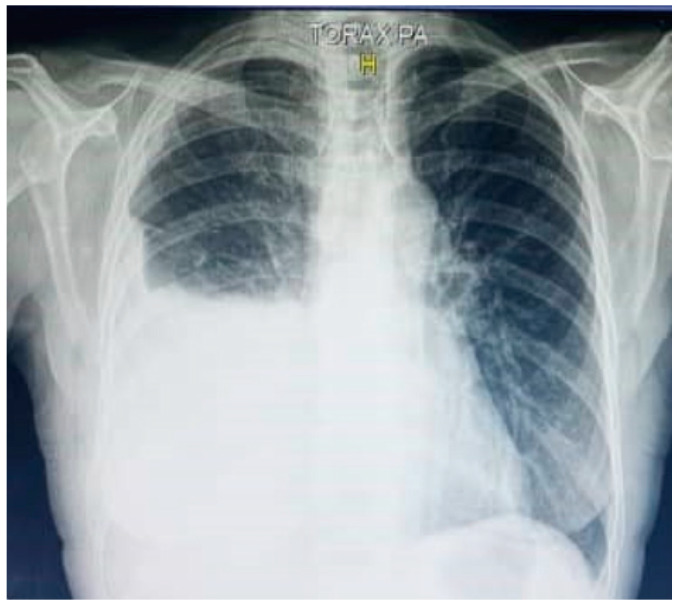
Posteroanterior chest X-ray with a typical lung collapse image of pleural effusion. A dense, homogeneous opacity at the lower two-thirds of the right hemithorax obscures the ipsilateral cardiac and diaphragmatic borders.

**Figure 2:**
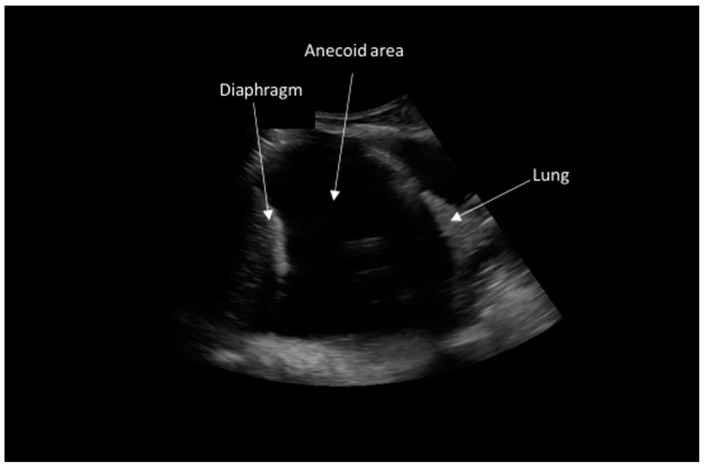
Pleural echography. The observation of an anechoic image is consistent with a pleural effusion pattern.

A 65-year-old woman presented with progressive dyspnea upon moderate exertion, which worsened over three weeks, along with a non-productive cough, asthenia, malaise, and night sweats, without a history of fever, weight loss and smoking. On physical examination, tachypnea with absent breath sounds in the right hemithorax was noted, and no lymphadenopathy was revealed by palpation. Chest X-ray revealed findings consistent with a pleural effusion occupying the lower two-thirds of the right hemithorax (Figure 1). In the lung ultrasound, an anechoic area devoid of internal echogenic structures was observed in the right hemithorax, confirming pleural effusion (Figure 2).

Initially, thoracentesis and pleural biopsy using an Abrams needle were performed. Subsequently, a chest tube was placed, and 2500 mL of turbid sero-fibrinous fluid was collected. Cytochemical analysis of the pleural fluid showed a pH of 9, LDH 92 IU/L, glucose 65 mg/dL, protein 3.7 mg/dL, cellularity 3400 cells/mm^3^, neutrophils 34%, lymphocytes 66%, compatible with a lymphocyte-predominant exudate. Adenosine deaminase (ADA) was 43 U/L, Ziehl–Neelsen and Grocott stains, cultures, GeneXpert MTB/RIF, and cytology were negative. A pleural biopsy revealed chronic granulomatous pleuritis without central necrosis.

Laboratory workouts showed a normal full blood count, metabolic panel, and hormonal profile. Renal and hepatic function tests were normal. Fasting blood glucose was 71 mg/dL, total protein 5.9 g/dL, lactate dehydrogenase (LDH) 102 IU/L, VDRL, and HIV were non-reactive. High-sensitivity C-reactive protein was 4.8 mg/L. Serology for cytomegalovirus and Epstein–Barr virus showed positive IgG. Antinuclear antibodies and tumour markers were negative.

### 2.1. Diagnostic Imaging and Video-Assisted Thoracoscopic Surgery (VATS)

**Figure 3:**
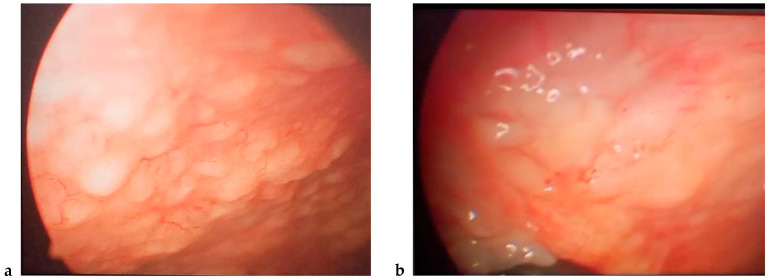
Video-assisted thoracoscopy. Multiple, irregular, and white-coloured nodules in the middle (**a**) and infefior (**b**) parietal pleura.

A high-resolution chest computed tomography (HRCT) scan demonstrated a right-sided pleural effusion with no evidence of lymphadenopathy or focal masses. Abdominal, pelvic, breast, and thyroid ultrasound scans were unremarkable. Transthoracic echocardiography showed preserved systolic function. Two months later, recurrent right pleural effusion with no definitive aetiology despite prior procedures and diagnostic studies prompted video-assisted thoracoscopy via a right fifth intercostal port. This procedure revealed a pleural cavity containing a significant volume of chylous fluid and multiple disseminated nodular lesions predominantly involving the parietal pleura (Figure 3).

### 2.2. Pathology

**Figure 4:**
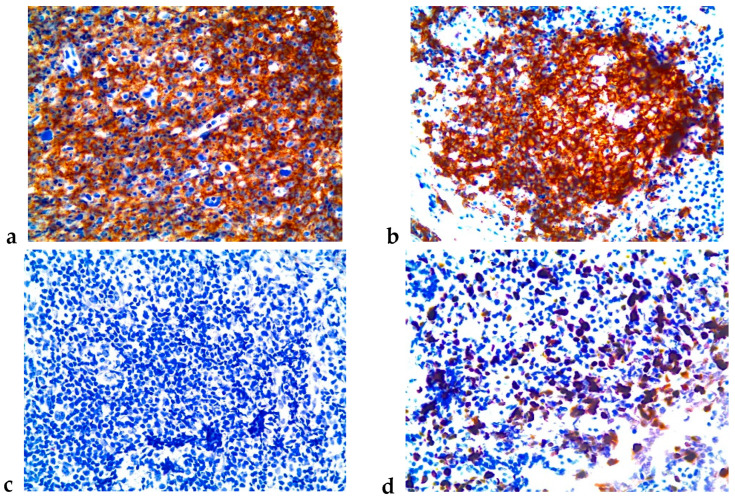
Immunohistochemistry. Deparaffinised tissue sections were subjected to antigen retrieval and incubated with monoclonal and/or polyclonal antibody panels. A biotin-free visualisation technique was employed using a peroxidase–polymer complex and DAB as a chromogen. (**a**) LCA (Leukocyte common antigen) (+); (**b**) CD20 (+); (**c**) CK (Cytokeratin) (−); (**d**) KI67 (+).

Visceral and parietal pleura biopsy revealed a dense, atypical lymphocytic infiltrate composed of large and medium-sized cells arranged in a mixed histological pattern. Immunohistochemistry demonstrated positivity for CD45/LCA, CD20/L26, PAX-5, and BCL-2. Ki-67 was expressed in 60% of lymphoid cells, while the immunostain was positive for CD30 in blastic immune cells. CD68 was positive in some macrophages and rare eosinophils. These immunohistochemical findings were consistent with diagnosing primary pleural B cell lymphoma with a mixed histological pattern (Figure 4).

The immunophenotypic profile was notable for the lack of expression of T-cell lineage markers (CD3-, CD45RO) within the tumour cells, with positivity restricted to background reactive lymphocytes. The neoplasm was uniformly negative for germinal centre (CD10-, BCL-6-, MUM-2-), mantle cell (CD5-, cyclin-D1-, SOX-11-), and lymphoblastic (TdT-) markers, as well as Hodgkin-associated antigens (CD15-, CD30-, fascin-), excluding differential diagnoses such as DLBCL, Hodgkin lymphoma, and blastoid variants.

### 2.3. Treatment and Follow-Up

**Figure 5:**
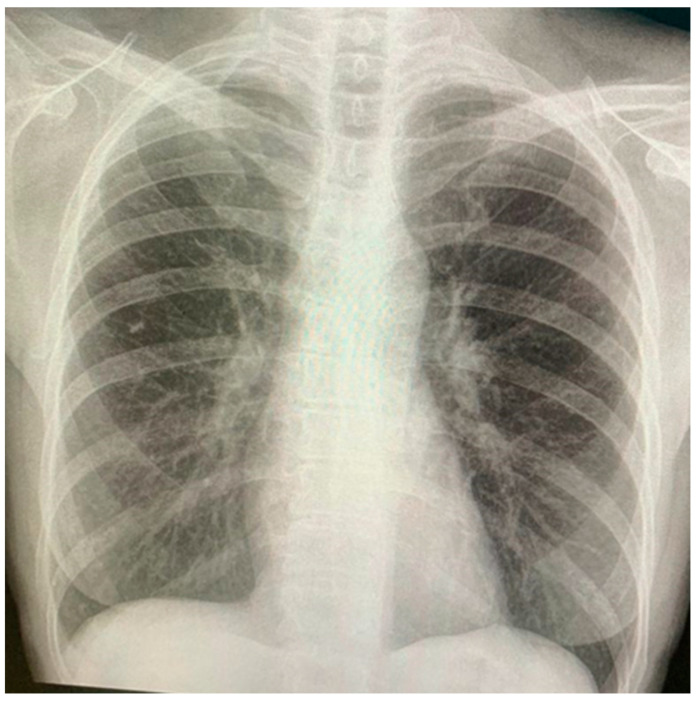
Posteroanterior chest X-ray seven months after the chemotherapy completion. Chest X-ray at posteroanterior projection. Well-positioned, well-inspired, and well-penetrated. Heart size is within normal limits. Lung fields are clear with no evidence of infiltrates. Hilar regions are unremarkable, and no pleural effusion was noted.

The patient received eight cycles of chemotherapy consisting of cyclophosphamide 750 mg, doxorubicin 50 mg, vincristine 1.4 mg, and dexamethasone 20 mg, combined with rituximab 375 mg every 21 days, with a significant clinical and radiological response. No respiratory or constitutional symptoms were reported during the one-year follow-up period. Seven months after the chemotherapy completion, a PET-CT scan showed no evidence of 18F-fluorodeoxyglucose (FDG) uptake. A follow-up chest X-ray one-year post-treatment revealed no evidence of pleural or pulmonary lesions (Figure 5). There was no recurrence of pleural effusion. Given the favourable outcome, the patient was maintained without further treatment, with continued clinical and radiological follow-up.

## 3. Discussion

Pleural effusion (PE) is a common clinical entity since an estimated 1.5 million patients in the United States experience PE annually. Although many causes exist, heart failure, infections, and cancer are the most frequent [25,26,27]. While pleural involvement is a common manifestation of secondary lymphoma, primary pleural lymphoma presents primarily as a PE [28], and due to the limited number of case reports, it is considered an extremely rare disease [1]. Two types of primary pleural lymphoma have been described in the medical literature: primary effusion lymphoma, mainly associated with the human immunodeficiency virus, and pyothorax-associated lymphoma, linked to human herpesvirus-8 and Epstein–Barr virus [10,12]. Most pleural lymphomas develop in association with preceding long-standing pleural diseases such as chronic tuberculous pyothorax or artificial pneumothorax for lung tuberculosis [29]. Given all of this, it should not be surprising that PPL has been primarily associated with immunosuppressed patients or chronic pleural inflammation [30], as it is extremely rare in immunocompetent patients or those without a history of pyothorax [31]. Chronic inflammatory stimulation is believed to lead to abnormal secretion of IL-6 and IL-10, promoting large malignant B cells to evade immune system surveillance. These interleukins help these cells, thus causing malignant proliferation. However, when suspected, early diagnosis through video-assisted thoracoscopy and biopsy is crucial, as prompt initiation of treatment is essential. Although not standardised, polychemotherapy is the ideal approach to improve outcomes [4,32].

As reported by other authors, the symptoms in our case were non-specific, presenting as a combination of constitutional symptoms and those characteristic of pleural effusion, such as dyspnea and cough, depending on the affected area and the severity of the effusion, making the PPL diagnosis based solely on clinical presentation a challenging task. Furthermore, the patient had no significant medical history in the present case. However, the extensive pleural effusion and nodular pleural lesions observed in imaging studies prompted a thoracoscopic pleural biopsy, which is essential for diagnosing primary pleural diffuse large B-cell lymphoma [1,4,33].

Pleural fluid analysis and cytology are rarely diagnostic for PPL and usually require an invasive procedure, such as video-assisted thoracoscopic surgery or image-guided pleural biopsy [34]. As previously mentioned, our patient had no history of systemic disease, chronic infections, immunological disorders, or previous pleural disease, as reported in some cases [28]. Only positive IgG antibodies for cytomegalovirus and Epstein–Barr virus were observed. Epstein–Barr virus has been strongly linked to lymphoma associated with pyothorax (pyothorax was not observed in this case). However, in sporadic cases, primary effusion lymphoma has been associated with this viral agent infection, as in this patient, where it could have induced prolonged pleural chronic inflammation [10,12]. A similar case was reported by Martin et al. in three immunocompetent patients, where Epstein–Barr virus-encoded small nuclear RNAs were detected by in situ hybridisation in most lymphoma cells in all cases [35]. This relationship was not demonstrated in our patient. Regrettably, viral RNA detection by hybridisation was not performed in our study, as our institution lacks the requisite equipment for this assay.

In the presence of isolated pleural effusion, as in our case, infections, pleural diseases, and lymphatic obstruction are frequent differential diagnoses [10]. Nevertheless, neoplasms, metastatic tumours, and pleural mesotheliomas are common differential diagnoses, especially when pleural thickening and nodules are present [3]. Due to the high prevalence rates of tuberculosis (TB) in our country, pleural tuberculosis is an important differential diagnosis among infectious pleural effusion causes. In this context, a lymphocyte-predominant pleural fluid exudate, positive ADA, and origin from a TB-endemic area suggested a differential diagnosis of TB pleurisy. However, negative acid-fast bacilli and GeneXpert results in pleural fluid, absence of caseating necrosis in granulomas, and negative Ziehl–Neelsen staining of the pleural biopsy ruled it out. In this regard, Cerezo-Hernández et al. reported similar characteristics in the pleural fluid of PPL associated with chronic empyema [20].

However, pleural TB is a frequent misdiagnosis when lymphoma presents with pleural effusion due to the high level of ADA in the pleural fluid. T cells can cause an elevation of ADA in tuberculous pleural effusion. Still, lymphoma’s pleural invasion also leads to more lymphocytes in the pleural effusion, which increases the ADA concentration. In this regard, some studies have shown that an increase in the concentration of ADA in the pleural effusion may indicate lymphoma. Current evidence suggests that pleural ADA levels in primary pleural lymphoma may be significantly elevated compared to other lymphoma subtypes and tuberculous pleuritis. Consequently, higher ADA values should raise stronger suspicion of lymphoma over tuberculosis in the diagnostic workup [36]. In our case, given that TB is a prevalent entity, the presence of ADA (adenosine deaminase) along with clinical–radiological features supported its consideration as one of the differential diagnoses.

Chest CT in primary pleural lymphoma has features like circumferential and nodular pleural thickening, mediastinal pleural involvement, and pleural effusion [37]. Occasionally, only isolated pleural effusion is present before a demonstrable mass, as in our patient who presented with a massive right-sided pleural effusion causing an ipsilateral lung volume decrease [38]. In this sense, video-assisted thoracoscopy and pleural biopsy are the gold standard recommended for diagnosing PPL [11,29]. In our case, video-assisted thoracoscopy allowed the visualisation of pleural nodular lesions and their subsequent biopsy.

Histological evidence is the final step for diagnosing this disease [10,39], and the appropriate markers support the diagnosis with specific immunophenotypic characteristics [40]. PPL generally lack expression of B-cell markers (CD19, CD20, CD79a, and PAX5) but frequently show lymphocyte activation markers (CD30, CD38, CD71) and plasma cell differentiation markers (CD138) [1]. PPL commonly exhibits a null lymphocyte phenotype (absent B- and T-cell markers). Cytogenetic analysis reveals complex karyotypes without consistent chromosomal abnormalities in PPL [1]. PPL cells typically express the common leukocyte antigen CD45 while demonstrating variable staining for plasma cell-associated markers. The absence of CD20 expression suggests limited therapeutic efficacy of anti-CD20 monoclonal antibody therapy, representing a significant treatment challenge in PPL [13]. However, CD45 and CD20 antigens were present in our case, expanding the therapeutic options. In many lymphoma subtypes, the advent of high-throughput sequencing methods, such as next-generation sequencing (NGS), significantly optimises mutational landscape identification [41]. However, in PPL, data remain scarce due to the rarity of this disease. Nevertheless, this research avenue holds considerable potential to refine diagnostic procedures, risk stratification, and patient monitoring. The diagnostic utility of genomic features in PPL and their impact on therapeutic decision-making remain unresolved and warrant further investigation through prospective clinical trials.

Although there is no standardised treatment for primary pleural lymphoma, all reports agree that chemotherapy is the best therapeutic strategy [4]. A combination of cyclophosphamide, vincristine, doxorubicin, dexamethasone, and rituximab every three weeks for eight cycles was used in this case. Similar treatment regimens using prednisone instead of dexamethasone every three weeks for six cycles with good responses have been reported. This regimen, known as R-CHOP, has demonstrated improved survival and progression-free outcomes in clinical trials [4,37,40]. Rituximab, a monoclonal antibody against CD20, has demonstrated advantages in B-cell lymphoma treatment because more than 95% of B-cell lymphomas express the CD20 antigen. Thus, CD20+ B lymphocyte clearance by antibody-dependent and complement-mediated cytotoxicity induces apoptosis of malignant B-cells [41]. In our patient, CD20 positivity provided a targeted and less toxic therapeutic alternative compared to standard chemotherapy. This finding supports CD20 expression as a favourable prognostic biomarker associated with improved overall survival in this pathology. Immunotherapy with rituximab and chemotherapy has shown better survival rates [42], highlighting that polychemotherapy improves survival and patient prognosis [4].

Over the past decade, novel anti-CD20 monoclonal antibodies have been developed, though their efficacy and safety profiles compared to rituximab remain controversial. Among second-generation anti-CD20 monoclonal antibodies, ofatumumab has been the most extensively studied and was approved by the FDA for treating relapsed/refractory chronic lymphocytic leukaemia (CLL). Third-generation agents include obinutuzumab and ocaratuzumab. Obinutuzumab has received FDA approval for CLL and follicular lymphoma (FL) treatment. However, clinical trial data remain limited, particularly regarding head-to-head comparisons with established therapies [43]. Because primary pleural lymphoma is rare, there are few studies to guide treatment. However, combination chemotherapy remains the main treatment [14], with CHOP as the standard regimen [44].

High-dose methotrexate combined with CHOP has demonstrated improved median survival; however, its clinical utility is limited by significant toxicity, particularly due to its propensity to accumulate in effusions [45]. Recent studies have examined adding rituximab and lenalidomide to CHOP [46]. In the case of non-responding lymphoma associated with the Epstein–Barr virus, labelecleucel has been used. Pandei et al. [14] reported a case where CHOP with a growth factor helped a patient with lymphoma linked to the human herpesvirus 8. Gastesi et al. suggested that both chemotherapy and radiotherapy can be used after surgery for primary pleural lymphoma. Chemotherapy alone can lead to complete remission in about 35% of cases [3], while radiotherapy effectively controls the disease locally before or after chemotherapy [20].

This case highlights the relevance of a personalised medicine approach in rare diseases such as primary pleural lymphoma. The choice of the R-CHOP regimen, which combines standard chemotherapy with rituximab—a monoclonal antibody targeting the CD20 antigen—was based on the tumour’s immunohistochemical properties, which showed CD20 positivity. This personalised treatment approach enhanced the clinical response and reduced side effects, both of which are crucial in elderly patients with restricted comorbidities. Furthermore, the absence of recurrence after one year of follow-up supports the effectiveness of this approach tailored to the patient’s characteristics. In this regard, Table 1 summarises recently reported cases of PPL, highlighting their clinical features, diagnostic workup, and treatment. Similar to our case, most patients were elderly, presented with pleural effusion, and were managed with the CHOP regimen.

The prognosis for these patients is generally poor, with overall survival ranging from 6 to 9 months and a 62% relapse rate following complete remission. Prognostic factors include disease extent at diagnosis, immune status, and lactate dehydrogenase levels [48]. These findings highlight the value of personalised medicine approaches incorporating individual patient characteristics and tumour cytogenetics predisposing the patient to severe disease manifestations, making early diagnosis and intervention crucial for favourable outcomes. Given the unpredictable clinical course of these patients, the implementation of a tailored, multidisciplinary follow-up program is essential for individuals with PEL undergoing chemoimmunotherapy regimens. A key advantage of such programs is fostering close clinician–patient relationships, enabling comprehensive monitoring of general health status, early detection of clinical deterioration, and timely management of severe adverse events [49].

In patients with malignancies such as lymphoma, anxiety and depression are frequent sequelae. Thus, multidisciplinary care programs must integrate psychological support throughout the disease trajectory to facilitate early identification of these conditions and provide timely mental health interventions [50]. Quality of life may fluctuate from diagnosis through survivorship, yet physical, social, and emotional sequelae often persist. Consequently, a deeper understanding of survivorship challenges and quality-of-life dynamics is imperative to inform practice modifications, enhance patient education, and design targeted behavioural interventions [51].

While pleural effusion is common in clinical practice, this uncommon case in an immunocompetent patient underscores the importance of considering primary pleural lymphoma in the differential diagnosis. Despite its extreme rarity, this entity demonstrates distinct clinical, immunohistochemical, and therapeutic features, warranting personalised management strategies for timely diagnosis and targeted treatment selection.

## 4. Conclusions

Primary pleural lymphoma is a rare disease with a difficult diagnosis requiring a personalised approach that combines advanced diagnostic techniques and targeted therapies because clinical and radiological characteristics are non-specific. Video-assisted thoracoscopy and biopsy with specific immunohistochemical characteristics are the gold standard for diagnosing this disease, and chemotherapy plus immunotherapy are excellent tools for improving survival and prognosis.

In this regard, targeted medicine enhances diagnostic accuracy and optimises therapeutic outcomes, particularly in rare diseases where standardised protocols may not be applicable. Future research should explore the role of genomics and immunotherapy in the personalised management of these patients, aiming to improve survival and quality of life further.

## Figures and Tables

**Table 1:** Summary of some clinical features in recent primary pleural lymphoma cases.

Author	Age and Gender	Primary Site	Other Structures Involvement	Cancer Type	Treatment, Outcome
Yang et al. [36]	49, male	Left pleura	Chest wall, presence of left pleural effusion	DLBCL	CHOP chemotherapy was administered with a significant delay. Deceased.
Rabadão et al. [30]	81, female	Right superior lobe pleura	Long-standing pleural effusion, heart Failure	DLBCL	Cyclophosphamide and prednisolone; remission.
Sun et al. [10]	73, male	Left pleura	Left pleural effusion	DLBCL	Cyclophosphamide, pirarubicin, vincristine, and prednisolone (CHOP) chemotherapy; remission
Ru et al. [31]	74, female	Right pleural cavity	Anemia, right-sided pleural effusion	Small B-cell lymphocytic lymphoma (SLL)	Surgery; remission
Farissi et al. [4]	79, NA	Pleura	Tumoral Epiduritis	DLBCL	Initial: Cyclophosphamide, Vincristine, and Prednisone + Subsequent R-CHOP21
Katkov [43]	66, male	Pleura	Right pleural Effusion	Low-grade B-cell lymphoma	The patient refused any further diagnostic tests, including PET/CT, as well as any treatment for lymphoma.
Gastesi et al. [11]	57, male	Pleura		Primary pleural Hodgkin’s lymphoma	Not reported
Onatsko et al. [47]	88, woman	Pleura	Recurrent bilateral pleural effusions	Pleural marginal zone lymphoma (PMZL)	Deceased
Elsayed et al. [45]	34, male	Pleura	Left hemithorax, consistent with a large pleural effusion	Plasmablastic lymphoma.	cyclophosphamide, doxorubicin, vincristine, and prednisolone (CHOP), Deceased
Pandey et al. [14]	76, male	Pleura	bilateral pleural effusion	Primary effusion lymphoma (PEL, non-Hodgkin’s lymphoma)	cyclophosphamide, doxorubicin, vincristine, and prednisone (CHOP)

## Data Availability

No new data were created or analyzed in this study. Data sharing is not applicable to this article.
